# A Facile and Scalable Hydrogel Patterning Method for Microfluidic 3D Cell Culture and Spheroid-in-Gel Culture Array

**DOI:** 10.3390/bios11120509

**Published:** 2021-12-10

**Authors:** Chengxun Su, Yon Jin Chuah, Hong Boon Ong, Hui Min Tay, Rinkoo Dalan, Han Wei Hou

**Affiliations:** 1 School of Mechanical and Aerospace Engineering, Nanyang Technological University, Singapore 639798, Singapore; chengxun001@e.ntu.edu.sg (C.S.); chuahy01@outlook.com (Y.J.C.); hongboon.ong@ntu.edu.sg (H.B.O.); tayhuimin@ntu.edu.sg (H.M.T.); 2 Interdisciplinary Graduate School, Nanyang Technological University, Singapore 639798, Singapore; 3 Lee Kong Chian School of Medicine, Nanyang Technological University, Singapore 308232, Singapore; rinkoo_dalan@ttsh.com.sg; 4 Endocrinology Department, Tan Tock Seng Hospital, Singapore 308433, Singapore

**Keywords:** hydrogel patterning, 3D cell culture, organ-on-a-chip, microarray, spheroid culture

## Abstract

Incorporation of extracellular matrix (ECM) and hydrogel in microfluidic 3D cell culture platforms is important to create a physiological microenvironment for cell morphogenesis and to establish 3D co-culture models by hydrogel compartmentalization. Here, we describe a simple and scalable ECM patterning method for microfluidic cell cultures by achieving hydrogel confinement due to the geometrical expansion of channel heights (stepped height features) and capillary burst valve (CBV) effects. We first demonstrate a sequential “pillar-free” hydrogel patterning to form adjacent hydrogel lanes in enclosed microfluidic devices, which can be further multiplexed with one to two stepped height features. Next, we developed a novel “spheroid-in-gel” culture device that integrates (1) an on-chip hanging drop spheroid culture and (2) a single “press-on” hydrogel confinement step for rapid ECM patterning in an open-channel microarray format. The initial formation of breast cancer (MCF-7) spheroids was achieved by hanging a drop culture on a patterned polydimethylsiloxane (PDMS) substrate. Single spheroids were then directly encapsulated on-chip in individual hydrogel islands at the same positions, thus, eliminating any manual spheroid handling and transferring steps. As a proof-of-concept to perform a spheroid co-culture, endothelial cell layer (HUVEC) was formed surrounding the spheroid-containing ECM region for drug testing studies. Overall, this developed stepped height-based hydrogel patterning method is simple to use in either enclosed microchannels or open surfaces and can be readily adapted for in-gel cultures of larger 3D cellular spheroids or microtissues.

## 1. Introduction

The overall success rate for phase I to phase III clinical trials is estimated to be 13.8%, and can be as low as 3.4% for oncology medication [1]. This translational failure is largely due to the reliance on in vivo animal testing and the lack of preclinical in vitro model that can accurately predict the drug responses in humans [2,3,4,5,6,7]. With advances in tissue engineering and microfluidics, more complex in vitro 3D cellular models, including spheroid cultures and organ-on-a-chip platforms, have been developed and increasingly used in recent years [8,9,10,11]. Spheroids offer higher biological complexity in terms of structural and functional properties by recapitulating the cell–cell interaction and tissue-like architecture [9,12].

They are typically cultured in suspension (e.g., the hanging drop method or round bottom 96-well plate), and lack a surrounding extracellular matrix (ECM), which plays a vital role in mediating instructive signals for cell polarization, retention, and mobilization [4,13,14]. On the other hand, microengineered organ-on-a-chip systems have been widely used to reconstitute key functional unit of human organs by precisely manipulating the fluid flow and control of 3D tissue structure and ECM microenvironments [5,10,11,15].

In any cell culture platforms, the incorporation of ECM/hydrogel patterning is important to establish (1) more physiological 2D cell monolayers on hydrogel surfaces, (2) 3D cell cultures using cell-laden hydrogels, and (3) co-cultures of multiple cell types by hydrogel compartmentalization to recreate complex 2D/3D tissue architecture. Classical surface-tension-based hydrogel patterning in microfluidics includes the use of micropillars [16,17,18,19], or narrow openings [20]. However, the intermittent physical barriers give rise to discontinuous cell–ECM interface, which may hamper cell–cell and cell–ECM communication, or subjects cells to differential biochemical and biophysical cues. 

To address these issues, several groups have developed innovative hydrogel patterning techniques to form continuous cell–ECM interface in enclosed microchannels using a phaseguide [21], recoverable elastic barrier [22], or suspended gel [23]. While a spheroid-in-gel culture can be achieved by patterning a spheroid-containing ECM using the above methods, they are limited by the inability to manipulate single spheroids and precisely control spheroid positions within the ECM in enclosed microchannels [24,25]. 

These lead to the need to adapt hydrogel patterning techniques on open chambers to accommodate single spheroid assays [26,27]. However, as spheroid formation is performed in cell suspension, this requires manual transferring of individual pre-formed spheroids into microfluidic devices for hydrogel encapsulation, which is laborious and prone to human error. Hence, there is an unmet need to develop a spheroid-in-gel culture platform that allows for the precise positioning of single spheroids and integrates spheroid formation and in-gel culture on a single device. 

Our group previously developed a hydrogel patterning method based on channel stepped heights and capillary burst valve (CBV) effect for blood vessel studies [28,29]. Herein, we further explore the versatility and scalability of the stepped-height based technique for 3D cell cultures in enclosed microchannels and spheroid-in-gel cultures in an open-channel microarray format. We first demonstrate hydrogel (Collagen I) patterning in a parallel lane configuration, which can be multiplexed using either one or two stepped height features. 

Next, we developed a microarray chip for spheroid-in-gel culture using a single step “press-on” hydrogel confinement method. The initial formation of breast cancer (MCF-7) spheroids was achieved using an on-chip hanging drop culture, following which each spheroid was directly encapsulated on-chip within individual hydrogel islands at the same location. Lastly, we co-cultured spheroids with endothelial cells (HUVEC) to form a vascular layer surrounding the spheroid ECM region and demonstrated cancer drug testing (Paclitaxel) in this model. Taken together, the developed hydrogel patterning method is easy to fabricate by standard photolithography and soft lithography, simple to use, and can be readily adapted for use in open channels for high-throughput 3D spheroid assays.

## 2. Materials and Methods

### 2.1. Device Fabrication

The device was fabricated using standard photolithography and soft lithography. In brief, polydimethylsiloxane (PDMS) prepolymer was mixed with curing agent (Dow Corning, Midland, MI, USA) at the ratio of 10:1 (*w*/*w*) and poured onto the patterned silicon wafer mold, degassed for 30 min and cured for 2 h at 75 °C. The PDMS slab was cut out and retrieved from the mold, following which a biopsy puncher was used to create the inlet and outlet for the device. For the lane configuration chips, the PDMS slab was plasma bonded to a glass slide using a plasma cleaner (PDC-002, Harrick Plasma Inc, Ithaca, NY, USA). The bonded device was kept at 75 °C overnight to enhance bonding and allow for hydrophobic recovery. Devices were sterilized by ultraviolet light for 30 min prior to on-chip cell culture experiments. 

Cell culture. Human Umbilical Vein Endothelial Cells (HUVEC) were maintained using Endothelial Cell Growth Medium-2 (EGM-2) BulletKit (Lonza, Basel, Switzerland) supplemented with 1% Penicillin-Streptomycin (P/S). Human Lung Fibroblasts (HLF) were maintained using FGM^TM^-2 Fibroblast Growth Medium-2 (FGM-2) BulletKit (Lonza). Human breast cancer cells (MCF-7) were maintained using Dulbecco’s Modified Eagle Medium (DMEM) (Gibco, Life Technologies, Carlsbad, CA, USA) supplemented with 10% Fetal Bovine Serum (FBS) (Gibco) and 1% P/S. The cells were maintained at 37 °C in a humidified 5% CO_2_ incubator and passaged using 0.25% trypsin with 1 mM EDTA (Gibco) upon confluency. Passage numbers 3 to 10 were used for HUVEC and HLF. 

### 2.2. On-Chip Cell Culture in Lane Format Chips

For cell seeding into the lane configuration chips, Collagen Type I (3 mg/mL) (rat tail, Corning, NY, USA) was prepared as previously described by Shin et al. [17], following which the gel was loaded into the chip and allowed to crosslink at 37 °C for 30 min. For HUVEC seeding into the one-lane hydrogel chip, the fluidic channels were coated with 50 μg/mL of Fibronectin (Sigma Aldrich, St. Louis, MO, USA) for 30 min at 37 °C prior to cell seeding. HUVEC were dissociated and resuspended to a concentration of 2 million cells per mL, following which the suspension was loaded into the fluidic channel and the HUVEC were allowed to grow until confluency. 

For HLF seeding into the three-lane hydrogel chip, HLF were dissociated and resuspended into Collagen I (3 mg/mL) to a concentration of 1 million cells per mL. The HLF-laden collagen was loaded into the two side hydrogel channels and allowed to crosslink at 37 °C for 30 min, following which the center hydrogel channel was filled with cell-free collagen. Lastly, the two fluidic channels were loaded with FGM-2 to replenish the cells. The chip was incubated at 37 °C for 2 days before fixation and imaging.

### 2.3. On-Chip Spheroid-in-Gel Culture in Microarray Chip

For spheroid-in-gel culture on the microarray chip, the PDMS surface was made hydrophilic by a 1-min plasma treatment using a plasma cleaner (PDC-002, Harrick Plasma Inc, Ithaca, NY, USA) to ensure even spreading of hydrogel onto the surface of the island. MCF-7 cells were dissociated and resuspended to a concentration of 1.7 million per mL. Collagen I was added to the suspension at 20 μg/mL to enhance spheroid formation. MCF-7 suspension was loaded onto each island (3 μL per island), following which the PDMS chip was inverted on top of a water reservoir for a hanging drop culture. 

For investigating the optimal number of cells and droplet height for successful on-chip formation of spheroid, the concentration and volume of the MCF-7 suspension were varied accordingly. The chip was maintained 37 °C for 2 days to allow for spheroid formation. In selected experiments, the MCF-7 cells were labelled with the Vybrant™ DiO Cell-Labeling Solution (Thermo Fisher, Waltham, MA, USA) prior to seeding onto the chip. Upon spheroid formation, the chip was subjected to media evaporation in the 37 °C incubator, following which Collagen I (3 mg/mL) or Matrigel (4 mg/mL, Corning, NY, USA) was loaded onto each island (2 μL per island) for in-place encapsulation of the spheroid. 

The chip was incubated at 37 °C for 30 min to allow for gelation of collagen and Matrigel, following which DMEM with 10% FBS was added into the fluidic channel. For co-culture with HUVEC, the channel was coated with polydopamine (1 mg/mL) for 30 min at 37 °C to facilitate HUVEC adhesion and growth as previously described [30,31,32]. The polydopamine solution was prepared by dissolving dopamine hydrochloride (Sigma Aldrich, St. Louis, MO, USA) in Tris-HCl buffer, pH 8.5. The channel was washed with 1× Phosphate Buffer Saline (PBS) three times, following which a suspension of HUVEC (1.5 million/mL) was loaded into the channel and allowed to grow for 2 days to reach confluency. The HUVEC were cultivated under static conditions.

### 2.4. Immunostaining, Drug Treatment and Live/Dead Assay

The chip was washed with 1× PBS and fixed with 4% paraformaldehyde (PFA) (Sigma Aldrich, St. Louis, MO, USA) for 15 min, following which the cells were stained with AlexaFlour 568 phalloidin (0.17 μm, Life Technologies, Carlsbad, CA, USA) and Hoechst 33342 (1 µg/mL, Life Technologies, Carlsbad, CA, USA) by incubating at room temperature for 45 min. For staining of HUVEC with VE-Cadherin, the cells were permeabilized with 0.1% Triton-X 100 in PBS for 15 min, washed three times with 1× PBS, and blocked with 0.5% bovine serum albumin (BSA) in PBS for 2 h at room temperature. 

Following that, the cells were stained with VE-Cadherin rabbit anti-human CD144 primary antibody (10 μg/mL, Enzo, Farmingdale, NY, USA) overnight at 4 °C. On the next day, the cells were rinsed with 0.1% BSA in PBS three times, and fluorescently labeled with AlexaFluor 488 goat anti-rabbit secondary antibody (20 μg/mL, Life Technologies, Carlsbad, CA, USA) for 4 h at room temperature. For live/dead assay of spheroids, the chip was washed with 1× PBS and stained with Calcein-AM (0.4 µM Life Technologies, Carlsbad, CA, USA), Propidium Iodide (PI) (2 µg/mL, Biolegend), and Hoechst-33342 (1 µg/mL, Life Technologies, Carlsbad, CA, USA) at 37 °C for 30 min. 

The cells/spheroids were then imaged using a fluorescence microscope (Nikon Eclipse Ti, Melville, NY, USA). For drug treatment, once HUVEC reach confluency, Paclitaxel (Thermo Fisher, Waltham, MA, USA) was added into the fluidic channels at concentrations of 100 and 500 nM, following which the chip was cultured for 3 days before a live/dead assay. Spheroid fixed with 4% PFA was used as negative controls. Fluorescence intensity of Calcein-AM and PI was analyzed using ImageJ and calculated according to Equation (1).

(1) $$I=\left(I_{t}-I_{b}\right)/A$$

where $I_{t}$ is the integrated intensity, $I_{b}$ is the background intensity, and A is the spheroid area. Spheroid area is determined by Hoechst staining. Quantification of spheroid area by Hoechst staining and F-actin staining was found to be similar (Figure S1 in Supplementary Materials). We estimated $I_{b}$ by measuring the mean gray value of regions without cells and multiplying this value to the area of image. The intensity of Calcein-AM was normalized to the untreated group of each experiment. The same set of imaging parameters including exposure time and focal plane was used when acquiring the images to minimize inaccuracies in the fluorescence intensity.

### 2.5. Endothelial Barrier Integrity Study

Endothelial barrier integrity study. To assess the endothelial barrier integrity after drug treatment in the spheroid-in-gel chip, Collagen I (3 mg/mL) and HUVEC were seeded into the chip as described in Section 2.3. Sub-confluent HUVEC were treated with Paclitaxel (100 and 500 nM) and cultured for 3 days prior to the barrier permeability test. Fluorescence images were taken before and after 1 h incubation with 10 μg/mL 70 kDa dextran conjugated with fluorescein isothiocyanate (FITC, Sigma Aldrich, St. Louis, MO, USA) loaded in the fluidic channels. Fluorescence intensities within the hydrogel island were expressed as the fold change (time 60 min over 0 min) and normalized to the untreated control.

## 3. Results

ECM/hydrogel can be confined in designated microchannel regions by the sudden expansion of channel height without any micropillars or microstructures [28]. Briefly, with a two-layered PDMS device and a stepped height feature at the intersection of a hydrogel channel and fluidic channel, the ECM introduced at the bottom layer is confined at the stepped height owing to the CBV effect conferred by the sudden channel expansion along the Z-axis. 

**Figure 1:**
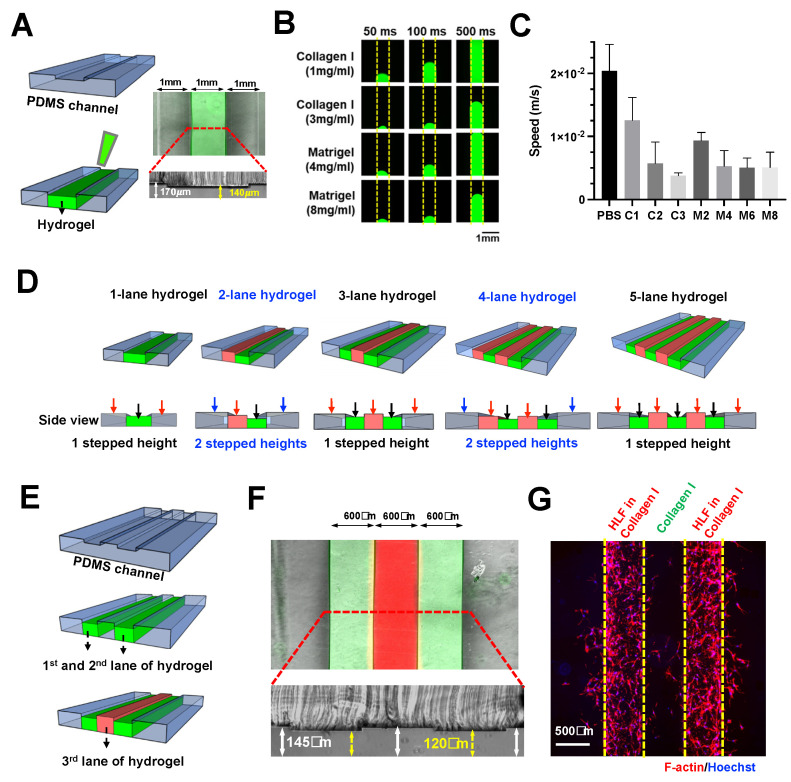
Scalable hydrogel patterning in enclosed microchannel using stepped height features. (**A**) Schematic illustration of stepped height-based hydrogel patterning in a single lane hydrogel chip. Overlaid fluorescence and brightfield image of the chip loaded with FITC-laden Collagen I (3 mg/mL) in the hydrogel channel (top). Cross-sectional view of the chip, white arrow indicates channel height of 170 μm, while yellow arrows indicate channel height of 140 μm (bottom). (**B**) Fluorescence images showing the gel loading process at different timepoints for Collagen I (1, 3 mg/mL, FITC-laden) and Matrigel (4, 8 mg/mL, FITC-laden). Yellow dotted line indicates the channel boundary. (**C**) Loading speed of 1× PBS, Collagen I (1, 2, and 3 mg/mL), Matrigel (2, 4, 6, and 8 mg/mL) in the one-lane hydrogel chip. (**D**) Schematic illustrating the concept of multi-lane hydrogel confinement using a single stepped height for odd number of lanes, and two stepped heights for even number of lanes. Black arrows indicate the first-layer channels, red arrows indicate the second-layer channels, and blue arrows indicate the third layer channels. (**E**) Schematic of sequential three-lane hydrogel loading. (**F**) Overlaid fluorescence and brightfield image of the chip loaded with FITC-laden Collagen I (3 mg/mL) in the first and third lanes, and R6G-laden Collagen I in the second lane (middle). Cross-sectional view of the chip, white arrow indicates channel height of 145 μm, while yellow arrows indicate channel height of 120 μm (bottom). (**G**) Fluorescence image of the chip containing two lanes of HLF-laden Collagen I with a cell-free Collagen I in between. (F-actin–red, Hoechst–blue).

Here, we further explored the scalability of this technique to pattern multiple (>2) adjacent lanes of hydrogel. Using a two-step photolithography method, a microfluidic device with a stepped height feature of ~30 μm was fabricated for single lane hydrogel confinement (Collagen I) within the shallower channel (middle) (Figures S2 and S3) (Figure 1A). 

Next, we investigated the patterning of different hydrogels by characterizing the loading speed for 1 × PBS, Collagen I (1, 2, and 3 mg/mL), and Matrigel (2, 4, 6, and 8 mg/mL). All the tested gel conditions were successfully confined in our device, thus, demonstrating the versatility of our technique. As expected, both collagen and Matrigel exhibited a concentration-dependent decrease in loading speed due to higher viscosities, which ranged from ~3.78 × 10^−3^ to 2.04 × 10^−2^ m/s (Figure 1B,C).

Micropillar-based multiple-lane hydrogel patterning has been reported by others for co-culture or modeling microenvironment [33,34]. Here, we demonstrated the use of stepped height for patterning multiple lanes of hydrogel. We propose that a single stepped height feature will be required for odd number of hydrogel lanes, while two stepped height features are needed for an even number of hydrogel lanes in a microfluidic design with fluidic channels at both sides (Figure 1D). 

To exemplify this, a three-lane hydrogel chip with a single stepped height (~25 μm) was fabricated (Figures S2 and S3). Sequential patterning of three hydrogel lanes (collagen mixed with red or green dye) was achieved (Figure 1E,F). The chip was used to demonstrate a 3D cell culture by patterning two Human Lung Fibroblasts (HLF)-containing hydrogel lanes with a cell-free hydrogel sandwiched in between them (Figure 1G), a platform, which is potentially useful for 3D cell migration assays.

We next adapted the method for single step “press-on” hydrogel confinement in an open-channel using a micropatterned PDMS substrate and glass slide. The PDMS substrate is first patterned with extruding stepped height features required for gel patterning to form individual “hydrogel islands”. Briefly, hydrogel droplets are deposited on the extruded features (island) of the open PDMS surface, following which the device is flipped over and pressed onto a glass slide. 

**Figure 2:**
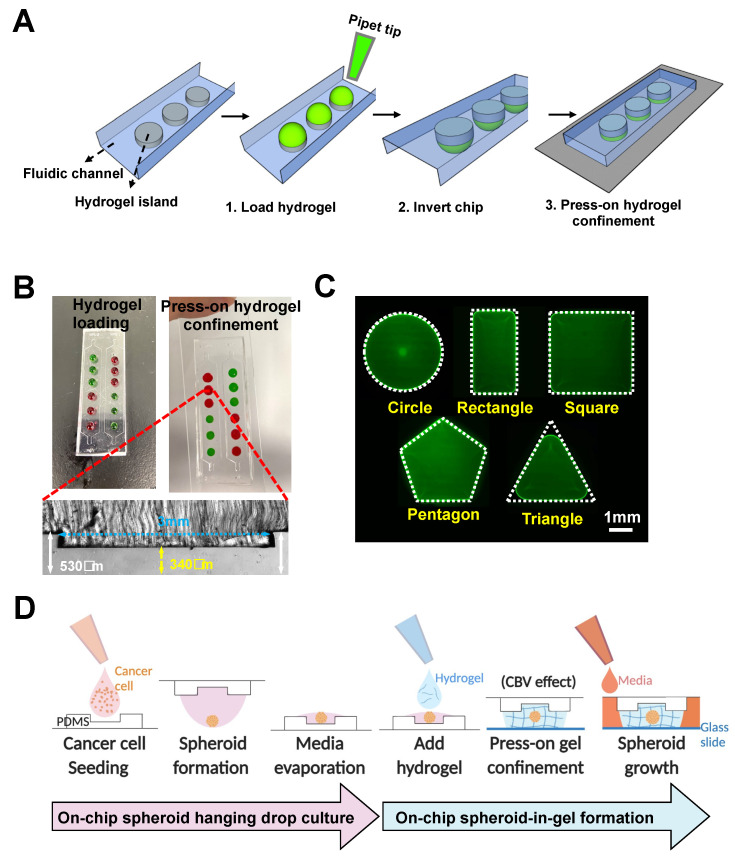
Rapid “press-on” hydrogel patterning in an open-channel microarray chip. (**A**) Schematic illustration of press-on hydrogel confinement on patterned islands on PDMS substrate. (**B**) Demonstration of gel loading and press-on gel confinement using Collagen I (3 mg/mL) mixed with red or green food dye (top). Cross-sectional image of the device, yellow arrows indicate the stepped height of ~190 μm (bottom). (**C**) Fluorescence images of FITC-laden Collagen I confined within different shapes of hydrogel island. White dotted line indicates the shape of the patterned hydrogel island. (**D**) Schematic illustration of workflow for on-chip spheroid-in-gel formation. Schematics were created with BioRender.com.

Upon contact, the hydrogel will remain confined within the island due to the surface tension resulted from the channel height differences (Figure 2A). For robust gel loading and press-on gel confinement, the channel heights for the main channel and hydrogel island were designed to be ~530 and ~340 µm, respectively, resulting in a step height of ~190 µm (Figure 2B). The deposited gel volume is calculated based on area of each island and channel height (~2.5 µL).

Successful gel loading and press-on gel confinement was demonstrated using Collagen I (3 mg/mL) mixed with food dye, and the user can spot different gels on each island as desired (Figure 2B). It should be noted that the gel loading process on open surfaces/channels is significantly easier compared with conventional gel patterning in enclosed microchannels as the user does not need to control the gel loading pressure, and the gel loading speed can be further increased with the use of electronic pipettes. 

To explore the versatility for hydrogel patterning, we first varied the shape of the hydrogel islands. Our results showed that hydrogel patterning was successful for different shapes, including circles, squares, rectangles, and pentagons, but was sub-optimal for shapes with angle of less than 60 degrees (e.g., equilateral triangles) (Figure 2C). For the equilateral triangle, a patterned hydrogel with a fillet radius of 0.6 mm was formed, which is likely due to the surface tension properties of the hydrogel itself. 

We next investigated the required dimensional parameters for circular islands, including the diameter and edge distance between each island. The circular island was selected for subsequent studies as it is isometric in nature and enables the formation of a concave surface (dome-shaped) that is similar to conventional hanging drop spheroid cultures. It was shown that hydrogel can be successfully patterned for circular diameters above 1.5 mm (Figure S4A), and adjacent patterned hydrogel did not spill over with an edge distance greater than 0.5 mm (Figure S4B). 

Additionally, we demonstrated that hydrogels (Collagen I, Matrigel, and Gelatin Methacryloyl) with different crosslinking mechanism (heat or UV) can be successfully patterned within the circular island (Figure S4C). A common approach to culture 3D cancer spheroid is the hanging drop method [35]. Briefly, a droplet of cancer cell suspension is spotted on a petri dish cover and inverted to allow for cell aggregation and spheroid formation by gravity in the concave surface. Here, we developed an on-chip hanging drop culture array using the extruded island features on a PDMS substrate. 

Although spheroid hanging drop culture can be performed on flat and hydrophobic PDMS surfaces [36], and the extruded islands are necessary to confer the stepped height to achieve hydrogel confinement due to the CBV effect upon contact with the glass substrate. Secondly, the spheroid-in-gel island array would facilitate co-culture of adherent cells (e.g., endothelial cells) outside the patterned islands in an enclosed channel.

Using our platform, a cancer cell suspension was directly loaded onto the extruded islands and remained confined as a droplet (Figure 2D). Upon spheroid formation in the hanging-drop culture, a portion of the culture media was removed by evaporation so that hydrogel can be deposited on the islands to encapsulate the spheroids. Once the hydrogel has crosslinked, culture media will be loaded into the channel and diffused through the hydrogel for spheroid culture. In addition to the extruded island feature, a centralized microwell was added to facilitate centralization of the spheroid in the developed spheroid-in-gel chip.

**Figure 3:**
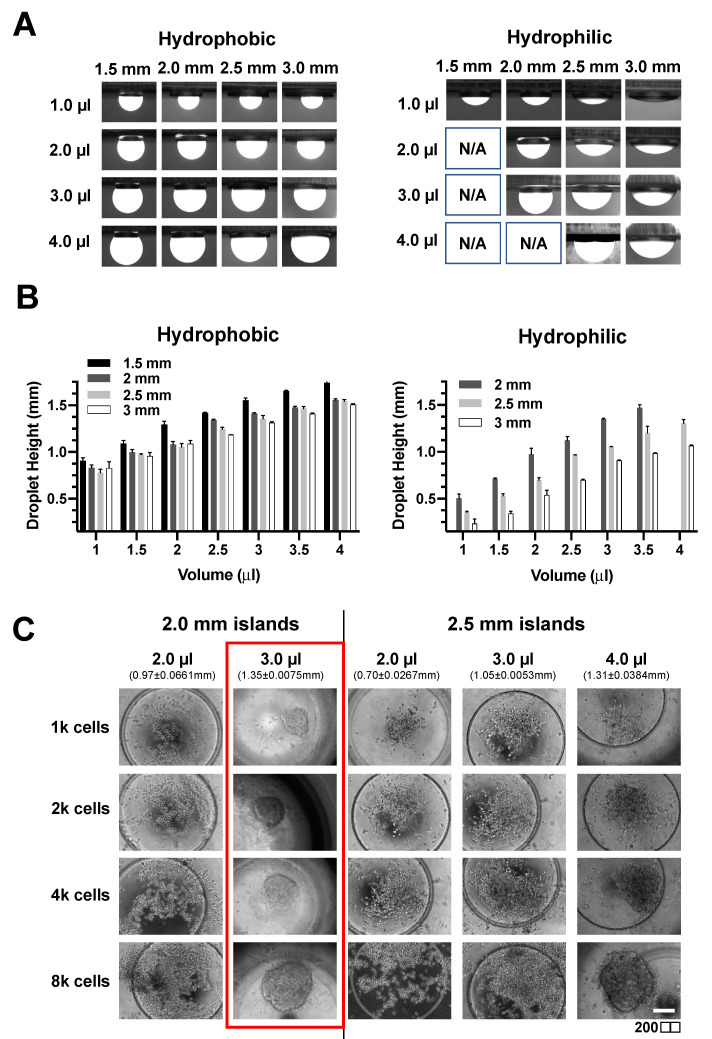
Optimizations for on-chip spheroid hanging drop culture. (**A**) Fluorescence images of FITC-containing droplets on hydrophobic and hydrophilic extruded circular island features. (**B**) Droplet height with different island diameter and volume of droplets on both hydrophobic and hydrophilic surfaces. Data was presented as mean ± SD. (*n* = 3) (**C**) Effect of cell number, droplet volumes, and island size on MCF-7 spheroid formation. Patterned islands consist of a microwell feature to facilitate spheroid centralization.

As the droplet shape will affect spheroid formation, we first investigated if the droplet height and contact angle can be tuned by varying the droplet volume, diameter of the circular island, and the surface hydrophobicity of PDMS. The patterned islands have an additional microwell feature to facilitate spheroid centralization during manual handling of the devices. While the hydrophobic surface allowed for droplet formation on islands of diameter greater than 1.5 mm for the four volumes investigated (1, 2, 3, and 4 μL), droplets were only formed on islands with a diameter above 2 mm for hydrophilic surface (Figure 3A). 

Hydrophilic surfaces also had lower droplet heights due to higher wettability (Figure 3B and Figure S5). Therefore, a hydrophilic surface was used for subsequent spheroid experiments due to the wider range of achievable droplet heights and contact angle (Figure 3B). Next, we investigated the number of cancer cells and droplet height for successful on-chip formation of spheroid using a breast cancer cell line (MCF-7). A defined amount of MCF-7 cell suspension was first loaded onto the island and inverted for a hanging drop culture for two days. Interestingly, it was observed that the formation of a spheroid required a minimal droplet height of 1.35 ± 0.0075 mm regardless of cell number (Figure 3C). Therefore, we set the volume as 3 μL of cell suspension on a circular island of a 2 mm diameter for optimal spheroid formation.

**Figure 4:**
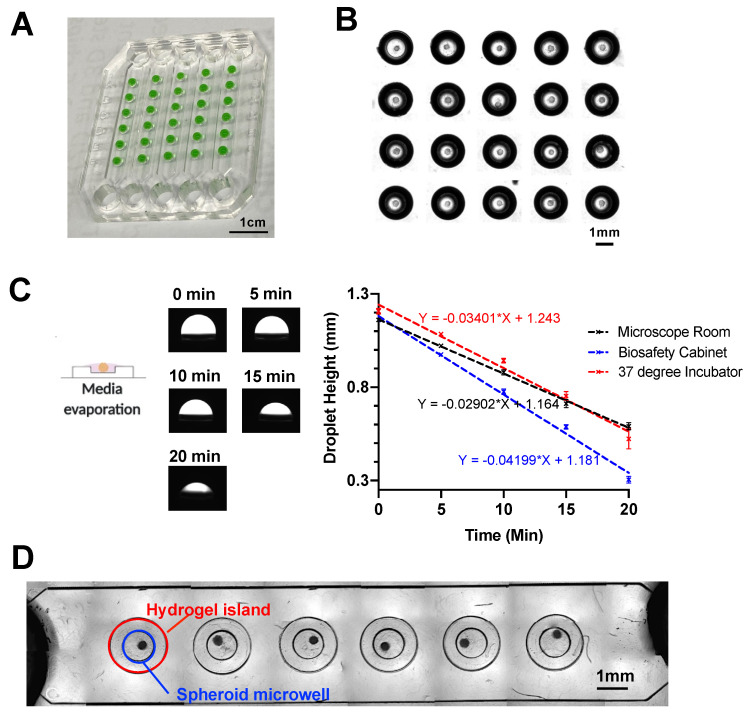
In-place encapsulation of spheroid on the microarray chip. (**A**) Image of water droplet (mixed with food dye) confined within a 5 × 6 microarray chip. (**B**) Stitched brightfield image of spheroids in hanging drop after culturing on chip for 2 days. (**C**) Evaporation rate of media droplet in three different environments. Data are presented as the mean ± SD. (*n* = 3) (**D**) Stitched brightfield image of a single channel of the 5 × 6 microarray chip with spheroids embedded within collagen gel. Red circle indicates the region of hydrogel island, and blue circle indicates the region of the spheroid microwell.

As a proof-of-concept for high throughput studies, the finalized design of spheroid-in-gel chip consists of five parallel main channels with each channel having six individual circular hydrogel islands (Figure 4A). The channel height for the main channel was ~930 μm, while the channel height for the microwell was ~820 μm to accommodate various sizes of spheroids (Figure S6). To combine the on-chip hanging drop method with stepped height-based hydrogel patterning on the same platform, it is important to remove the culture media on each island prior to addition of hydrogel. 

Upon successful spheroid formation after two days’ hanging drop culture (Figure 4B), we examined the media evaporation rate in three different environments, including the 37 °C 5%-CO_2_ incubator, biosafety cabinet and microscope room (at room temperature). After 20 min, all three environments showed a decline of droplet height to at least 50%, with biosafety cabinet having the highest evaporation rate possibly due to additional convective air flow (Figure 4C). 

The evaporation step was important to remove a portion of the culture media before adding the hydrogel to prevent spillage out of the island features. The process was also carefully performed to ensure the spheroids remained contained within the culture media to minimize any adverse effects. It should be noted the transient increase in concentration of soluble factors in the media may affect cell metabolism, which warrants further investigations.

To ensure minimal damages to the cultured spheroids, media evaporation was performed in the 37 °C incubator for the rest of the study. Next, Collagen I (2 μL) was added onto each island to encapsulate the spheroid, following which the “press-on” gel patterning was used to enclose the channel and fixate the spheroid-containing collagen against a glass slide. 

Spheroids were confined within the microwell region as intended (Figure 4D). As the spheroid position may vary within the hydrogel islands, a diffusion study was performed to investigate whether the spheroid position would affect the uptake of molecules. By incubating the spheroids with FITC (~400 Da) and FITC-10kDa Dextran for 24 h, we observed effective diffusion and uptake of both molecules into the spheroid regardless of the spheroid position (Figure S7).

Next, we investigated the feasibility to perform co-culture of spheroid with endothelial cells to recapitulate the vascular barrier surrounding the tumor as observed in vivo. For cell co-cultures, it is important to ensure that different cells are properly replenished using a common culture media. As such, we introduced different culture media (DMEM (for cancer cells), EGM-2 (for endothelial cells), and DMEM + EGM-2 (1:1)) at day 4 and examined the spheroid viability at day 7. We observed that the spheroids showed minimal cell death over a period of 7 days for all the three media tested (Figure S8). To ensure optimal growth of endothelial cells, the EGM-2 media was used for co-cultures of spheroid and endothelial cells [27]. 

**Figure 5:**
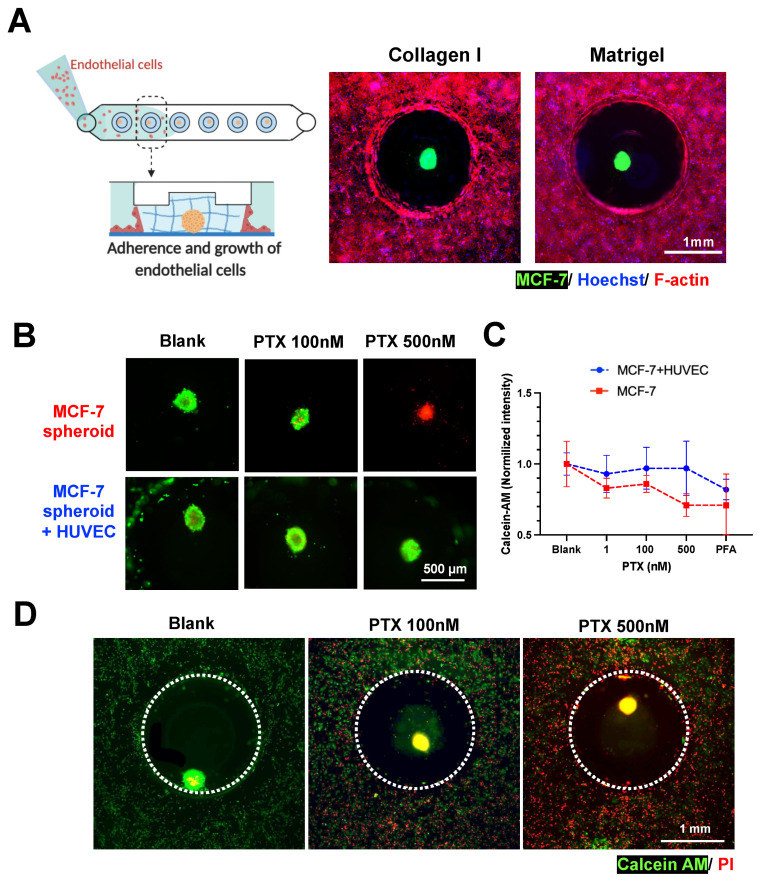
Spheroid-in-gel formation and co-culture with endothelial cells. (**A**) Co-culture of MCF-7 spheroids with endothelial cells (HUVEC) in collagen and Matrigel. MCF-7 was labelled with DiO, F-actin—red, and Hoechst—blue. Schematics were created with BioRender.com. (**B**) Merged fluorescent images illustrating viability of spheroid after PTX treatment. (Calcein-AM—green and PI—red) (**C**) Normalized fluorescence intensity of Calcein-AM for spheroids with or without HUVEC co-culture after 3 days of treatment with PTX 1, 100, and 500 nM. A PFA-fixed spheroid was used as negative control. Data are presented as the mean ± SD. (*n* = 3) (**D**) Merged fluorescent images illustrating viability of spheroids and HUVEC in co-culture after 3 days of PTX treatment. (Calcein-AM—green and PI—red).

Human umbilical vein endothelial cells (HUVEC) were introduced into the fluidic channel to form a monolayer at the channel bottom and along the gel surface of the MCF-7 spheroid-in-gel islands. As expected, a confluent layer of HUVEC was formed surrounding the spheroid ECM region after 2 days (Figure 5A and Figure S9), with the presence of an adherens junction marker (VE-Cadherin) (Figure S10). This shows the feasibility to establish a spheroid-in-gel co-culture using our platform. 

As a proof-of-concept for drug screening applications, the spheroids with or without HUVEC co-culture were treated with Paclitaxel (PTX) for 3 days, following which the viability of the spheroids were examined. Live/dead staining (Calcein-AM/PI) showed higher MCF-7 death with increasing drug concentration for mono-cultured spheroids, and higher EC death for spheroid co-cultured with HUVEC (Figure 5B,D). The quantification of spheroid Calcein-AM intensity further showed a trend of better survival of spheroids in co-cultures as compared to mono-culture, although no significant difference was observed. 

This is possibly due to presence of endothelial barrier or paracrine effects by the surrounding HUVEC, thus suggesting the importance of performing drug screening studies in physiological microenvironment (Figure 5C,D). Live/dead staining (Calcein-AM/PI) showed higher MCF-7 and EC death with increasing drug concentration (Figure 5B,D). Quantification of spheroid Calcein-AM intensity showed a trend of better survival of spheroids in co-cultures as compared to mono-culture (Figure 5C), although no significant difference was observed. 

There were also negligible differences in PI intensity between drug-treated spheroids in mono-culture and co-culture (Figure S11), which could be due to high red background noise and limited sensitivity with the 2D imaging method used. Finally, the increased EC death (Figure 5D) also impaired the endothelial barrier integrity based on the diffusion of FITC-Dextran 70kDa into the hydrogel islands (Figure S12). This barrier breakdown may affect spheroid–immune cell interactions if leukocytes are present in the culture platform, thus, suggesting the importance of adding an immune component to better model the tumor microenvironment as future work.

As the PDMS substrate can be separated from the glass slide due to reversible bonding in the spheroid-in-gel platform, we demonstrated that individual spheroid-containing gels can be retrieved using a tweezer manually (Figure S13). We envision that this unique feature of selected spheroid and ECM retrieval will be important for downstream immuno-oncology studies to characterize both tumor and immune/perivascular cells within the ECM.

## 4. Discussion

In this study, we developed a stepped height-based hydrogel patterning technique to create different chip configurations for 3D cell cultures in enclosed microchannels and spheroid-in-gel cultures in open channels. The stepped height-based method described here uses standard photolithography to fabricate a wafer mold with multi-height patterned features and a single-step PDMS soft lithography to obtain the final device. Therefore, it can be easily adopted by research laboratories where standard photo lithography and soft lithography are widely used and easily accessible. 

While the dual-lane stepped-height based hydrogel chip have been reported for arterial wall-on-a-chip [29], we further demonstrated the scalability of this method to pattern multiple adjacent lanes of hydrogel using single or dual stepped heights. An injection-molded culture platform for patterning three adjacent lanes of hydrogel have been reported [37], but it lacks the flexibility to pattern two different hydrogels for the first two lanes. 

Additionally, the height of the side hydrogel lanes and the center one differs by 15-fold, which can potentially hamper cell–cell or cell–ECM interaction between different hydrogel lanes. Here, as the stepped height features are located at the upper PDMS channel, this minimizes technical issues related to the imaging of a cell monolayer at the channel bottom and further facilitates the integration of electrode sensors at the bottom substrate for real-time biosensing capabilities in organ-on-chip devices. 

Secondly, we demonstrated that this stepped height-based hydrogel patterning method can be scaled-up in dimensions and adapted for operation in open channels to establish 3D spheroid-in-gel culture arrays. The culture platform demonstrated in this study was designed to have five main channels with each channel containing six islands for spheroid cultures. The design is easily amenable and can be scaled up as needed. Although a PDMS-based hanging drop culture platform without hydrogel patterning features has been reported, the culture system cannot be enclosed after spheroid encapsulation, and hence it is not possible to perform perfusion cultures or co-cultures of spheroids with other cell types [36]. 

Here, using a simple and rapid press-on hydrogel confinement method, we were able to achieve the following unique features including (1) versatility to pattern different hydrogel island shapes, (2) integrating on-chip spheroid formation and in-place gel encapsulation, which circumvents the need to manually transfer each spheroid into the gel, (3) flexibility to perform co-culture with other cell types, and (4) the retrieval of individual spheroid for off-chip downstream analysis.

As shear forces play an important role in endothelial functionality, we demonstrated that the hydrogel islands will not dislodge under flow (at a flow rate up to 500 μL/min for 5 min) in our developed platform (Figure S14), thus, suggesting the potential to perform perfusion culture as future work. Another important future work is to distinguish the structure of the 3D spheroids in hydrogel islands using confocal imaging. Lastly, while we demonstrated a toxicity effect after 3 days of drug treatment in this study, it is of importance to perform long-term monitoring of spheroid drug responses in the future.

Taken together, the spheroid-in-gel culture platform offers compelling advantages over existing spheroid culture platforms owing to its simple fabrication and operation steps as well as the ability to preserve the biological complexity needed for physiological relevancy while remaining scalable for high throughput studies. We envision that the developed hydrogel patterning technique and platforms would be of great interest for both fundamental studies and translational research, such as high content drug screenings.

## 5. Patents

A patent application titled “Micropatterned 3D hydrogel microarray in fluidic channels for spheroid-in-gel culture” resulting from this work was filed on 3 May 2021 (Singapore Patent Application No. 10202104559S).

## Data Availability

All data presented in this study are available in the article and Supplementary Material.

## Supplementary Materials

The following are available online at https://www.mdpi.com/article/10.3390/bios11120509/s1. Figure S1: Comparison of spheroid area measured by F-actin signal (red) and Hoechst signal (Blue). White dashed circle indicates channel boundary. Spheroids were embeded in Collagen I (3mg/mL). Figure S2: Fabrication method for the 1-lane hydrogel chip, 3-lane hydrogel chip, and press-on hydrogel microarray chip. Figure S3: Image of the 1-lane hydrogel chip (left) and 3-lane hydrogel chip (right). Figure S4. (A) Collagen I (3 mg/mL, containing 10 mM FITC) confined within circular islands of different diameter. (B) Different edge distance between two adjacent circular islands patterned with Collagen I (3 mg/mL, containing 10 mM FITC). (C) Hydrogel (containing 10 mM FITC) with different crosslinking mechanisms confined within the patterned circular island. Figure S5: Contact angle with different island diameter and volume of droplets on both hydrophobic and hydrophilic surfaces. Data was presented as mean ± SD. (n = 3). Figure S6: Cross-sectional view of one island on the microarray chip for spheroid-in-gel culture. Figure S7: FITC/FITC-10kDa Dextran (0.1µM) uptake into MCF7 spheroid co-cultured with HU-VEC in Collagen I (3mg/mL) and Matrigel (4mg/mL). After 24h incubation, channels were washed and fixed in 4% PFA for imaging. White dashed circle indicates the channel boundary. Figure S8: Viability of spheroids after hydrogel encapsulation and culturing in different media (from day 4) on day 7 (Calcein-AM – green, PI – red). Figure S9: Reconstructed 3D fluorescence image of HUVEC layer surrounding the ECM region (Collagen I, 3 mg/mL) on one island of the chip. (F-actin – red, Hoechst – blue). Figure S10: Fluorescence image of endothelial cells cultured in the spheroid-in-gel chip (VE-Cad – green, Hoechst – blue, F-actin – red). Figure S11: Normalized fluorescence intensity of PI for spheroids with or without HUVEC co-culture after 3 days of treatment with PTX 1 nM, 100 nM, 500 nM. PFA-fixed spheroid was used as negative control. Data was presented as mean ± SD. (n = 3). Figure S12: Normalized fluorescence intensity within the hydrogel island for untreated control, 100 nM and 500 nM Paclitaxel (PTX) treated HUVEC. FITC-dextran 70 kDa (10 μg/mL) was load-ed into the chip and allowed to diffuse into the island for 60 min before images were taken and analyzed. The fluorescence intensity was expressed as fold change (time 60 min over 0 min), and normalized to the mean of untreated control. Results were as expressed as mean ± SD, **p < 0.005. Figure S13: Retrieval of spheroid-laden hydrogel using forceps and resuspension in a microtube. Spheroid (small white dots) can be observed with naked eyes and are highlighted with red arrows. Figure S14: Stability of Collagen I (3 mg/mL) and Matrigel (4 mg/mL) at various flow rate. Hy-drogel were mixed with fluorescent microbeads for visualization. Images were taken after 5 min of perfusion.
