# Supplementary material for: A Facile and Scalable Hydrogel Patterning Method for Microfluidic 3D Cell Culture and Spheroid-in-Gel Culture Array
Source: Biosensors (Basel). 2021 Dec 10;11(12):509. doi: 10.3390/bios11120509 (PMC8699815; doi:10.3390/bios11120509)
Supplement: Supplementary file 1 [file biosensors-11-00509-s001.zip › biosensors-1445406-supplementary.pdf]

biosensors

Article

A Facile and Scalable Hydrogel Patterning Method for Microfluidic 3D Cell Culture and Spheroid-in-Gel Culture Array

Chengxun Su [1,2], Yon Jin Chuah [1], Hong Boon Ong [1], Hui Min Tay [1], Rinkoo Dalan [3,4] and Han Wei Hou [1,3,*]

[1] School of Mechanical and Aerospace Engineering, Nanyang Technological University, Singapore 639798, Singapore; chengxun001@e.ntu.edu.sg (C.S.); chuahy01@outlook.com (Y.J.C.); hongboon.ong@ntu.edu.sg (H.B.O.); tayhuimin@ntu.edu.sg (H.M.T.)
[2] Interdisciplinary Graduate School, Nanyang Technological University, Singapore 639798, Singapore
[3] Lee Kong Chian School of Medicine, Nanyang Technological University, Singapore 308232, Singapore; rinkoo_dalan@ttsh.com.sg
[4] Endocrinology Department, Tan Tock Seng Hospital, Singapore 308433, Singapore
* Correspondence: hwhou@ntu.edu.sg

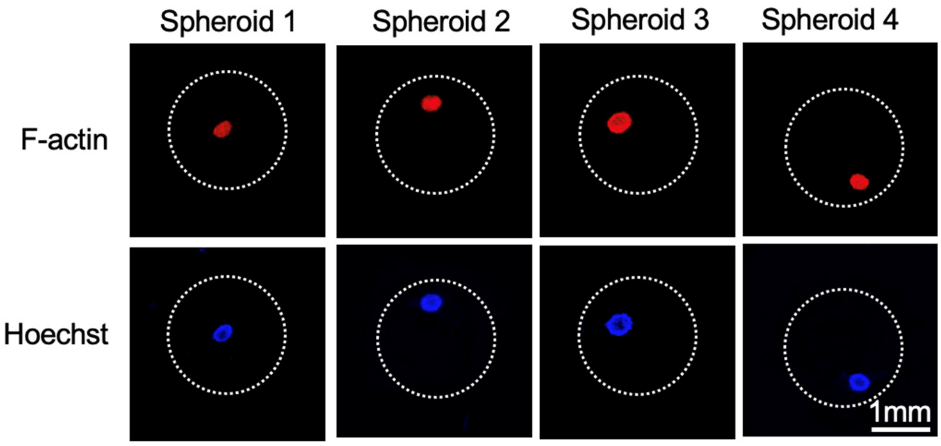

Spheroid	Area (um^2, by F-actin)	Area (um^2, by Hoechst)	%CV
1	66851.967	73940.675	5.0%
2	77757.671	84519.208	4.2%
3	113746.495	118981.233	2.2%
4	75576.53	83537.694	5.0%

Figure S1. Comparison of spheroid area measured by F-actin signal (red) and Hoechst signal (Blue). White dashed circle indicates channel boundary. Spheroids were embeded in Collagen I (3mg/mL).

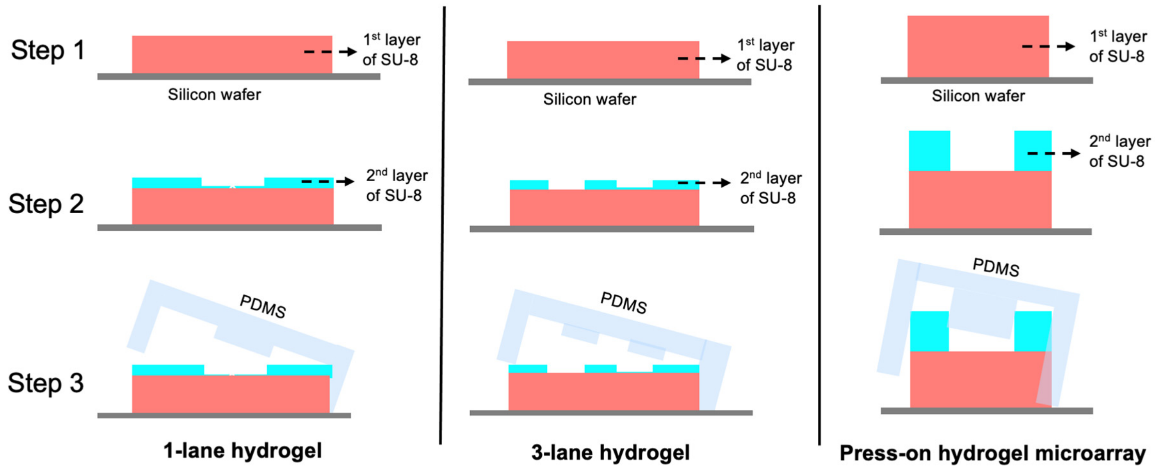

Figure S2. Fabrication method for the 1-lane hydrogel chip, 3-lane hydrogel chip, and press-on hydrogel microarray chip.

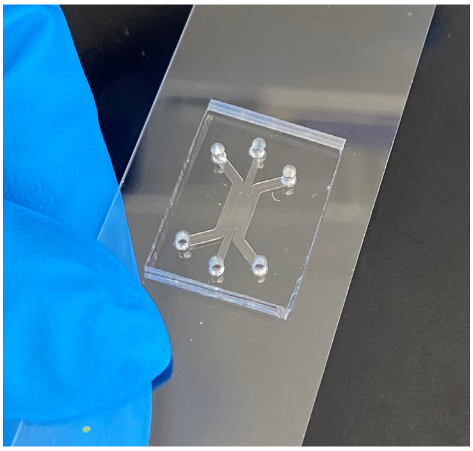
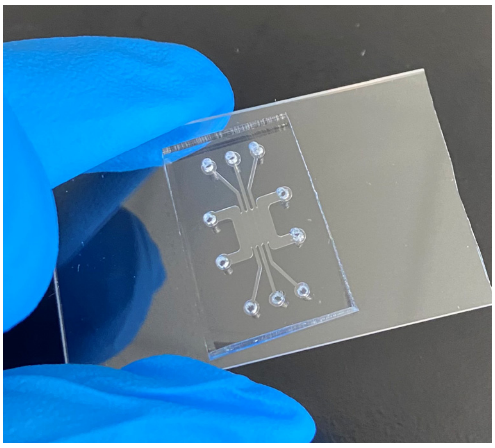

Figure S3. Image of the 1-lane hydrogel chip (left) and 3-lane hydrogel chip (right).

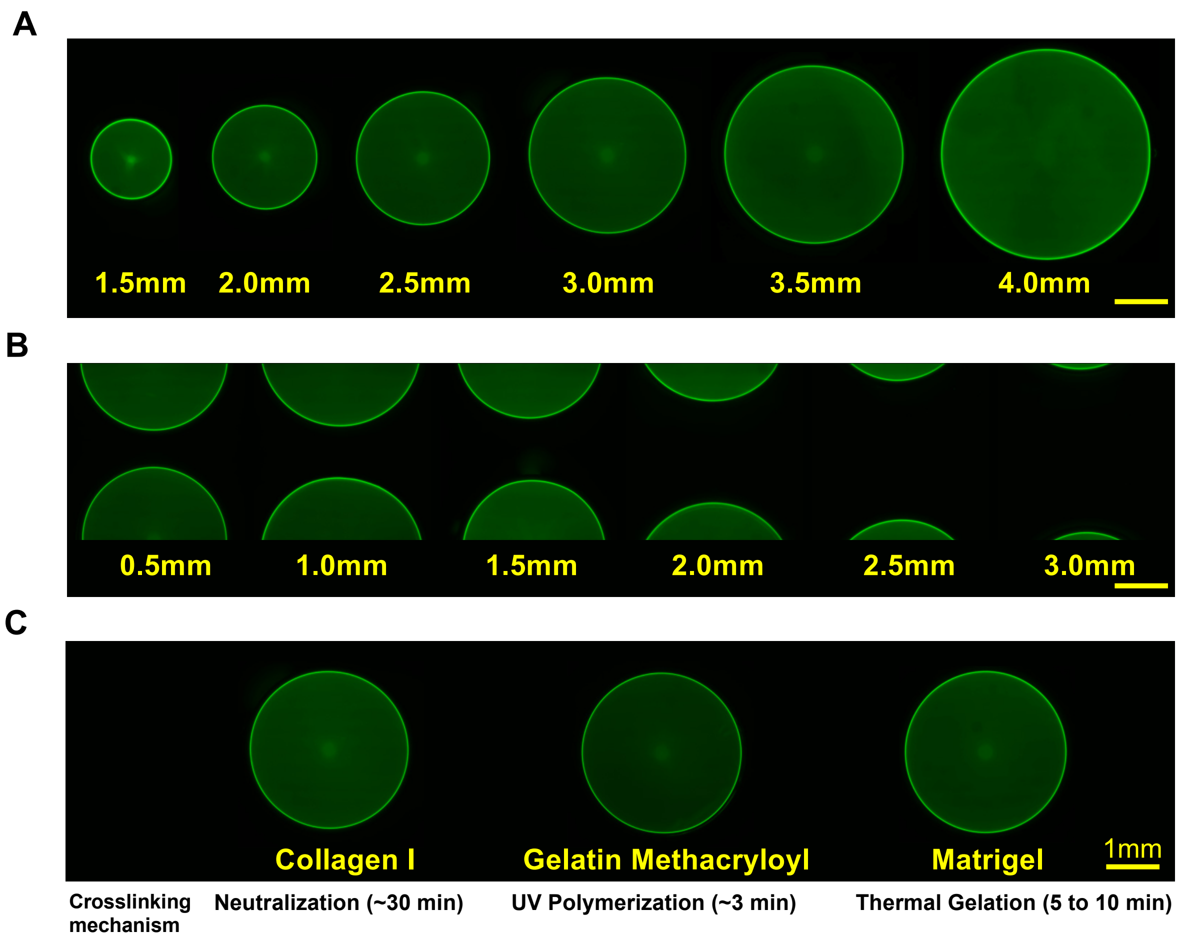

Figure S4. (**A**) Collagen I (3 mg/mL, containing 10 mM FITC) confined within circular islands of different diameter. (**B**) Different edge distance between two adjacent circular islands patterned with Collagen I (3 mg/mL, containing 10 mM FITC). (**C**) Hydrogel (containing 10 mM FITC) with different crosslinking mechanisms confined within the patterned circular island.

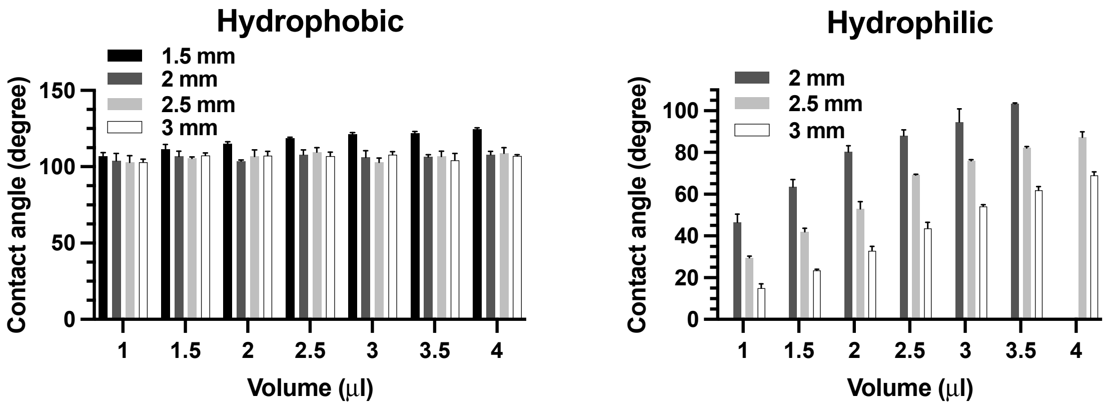

Figure S5. Contact angle with different island diameter and volume of droplets on both hydrophobic and hydrophilic surfaces. Data was presented as mean ± SD. (n = 3).

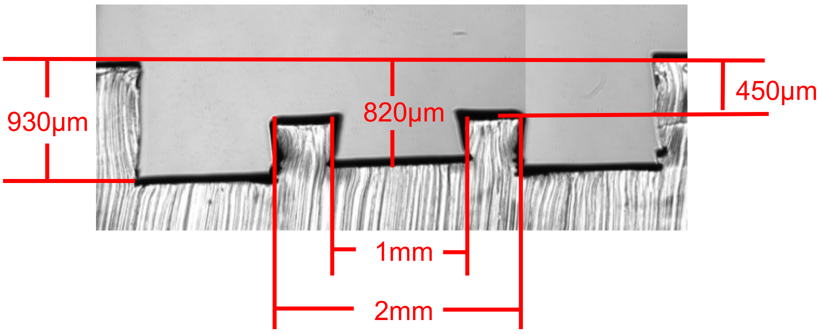

Figure S6. Cross-sectional view of one island on the microarray chip for spheroid-in-gel culture.

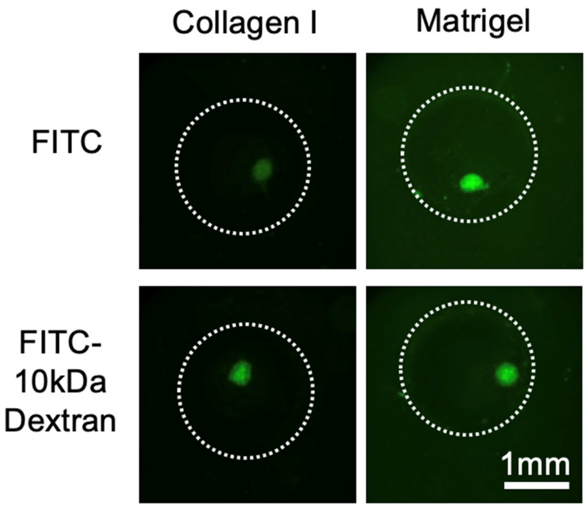

Figure S7. FITC/FITC-10kDa Dextran (0.1µM) uptake into MCF7 spheroid co-cultured with HUVEC in Collagen I (3mg/mL) and Matrigel (4mg/mL). After 24h incubation, channels were washed and fixed in 4% PFA for imaging. White dashed circle indicates the channel boundary.

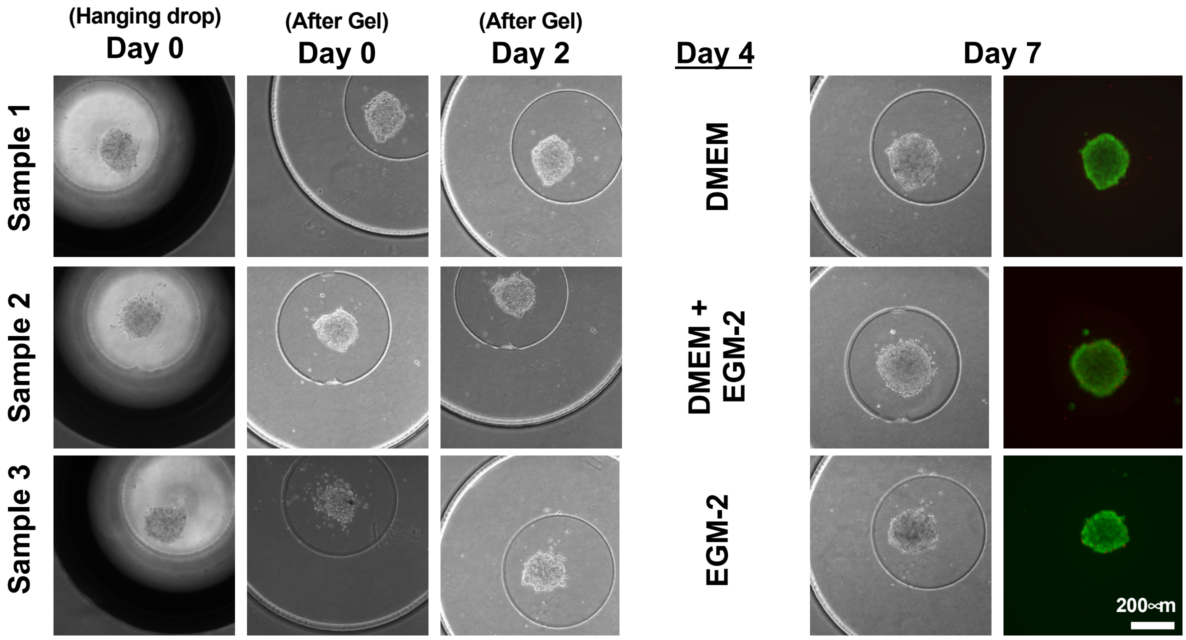

Figure S8. Viability of spheroids after hydrogel encapsulation and culturing in different media (from day 4) on day 7 (Calcein-AM – green, PI – red).

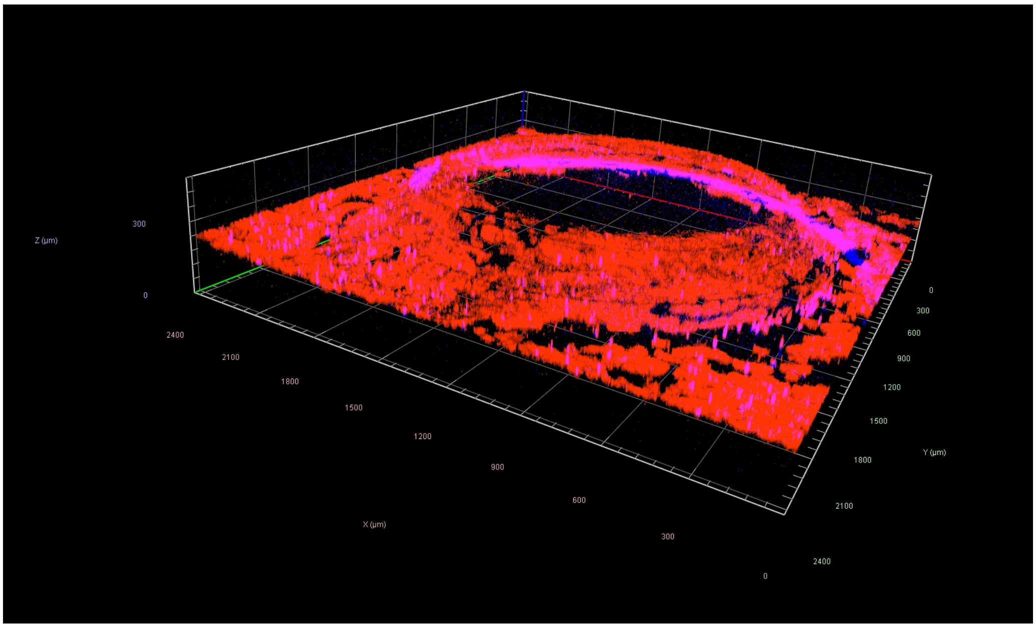

Figure S9. Reconstructed 3D fluorescence image of HUVEC layer surrounding the ECM region (Collagen I, 3 mg/mL) on one island of the chip. (F-actin – red, Hoechst – blue).

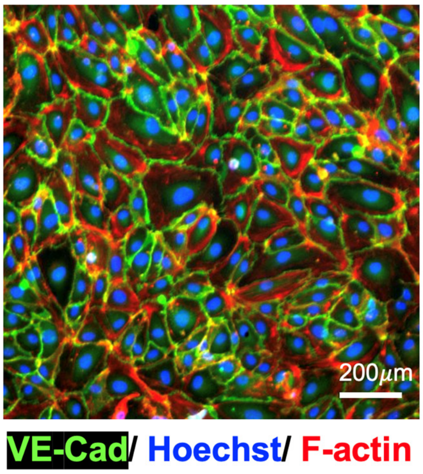

Figure S10. Fluorescence image of endothelial cells cultured in the spheroid-in-gel chip (VE-Cad – green, Hoechst – blue, F-actin – red).

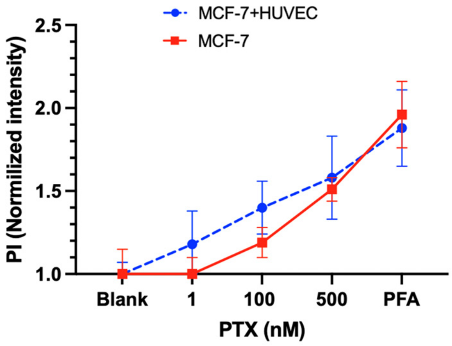

Figure S11. Normalized fluorescence intensity of PI for spheroids with or without HUVEC co-culture after 3 days of treatment with PTX 1 nM, 100 nM, 500 nM. PFA-fixed spheroid was used as negative control. Data was presented as mean ± SD. (n = 3).

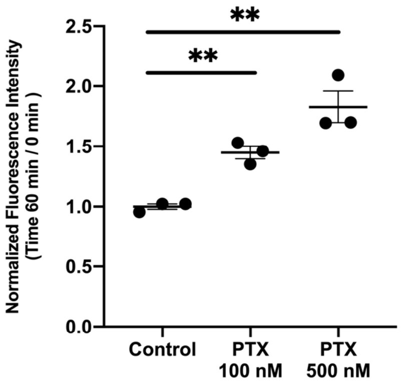

Figure S12. Normalized fluorescence intensity within the hydrogel island for untreated control, 100 nM and 500 nM Paclitaxel (PTX) treated HUVEC. FITC-dextran 70 kDa (10 μg/mL) was loaded into the chip and allowed to diffuse into the island for 60 min before images were taken and analyzed. The fluorescence intensity was expressed as fold change (time 60 min over 0 min), and normalized to the mean of untreated control. Results were as expressed as mean ± SD, **p < 0.005.

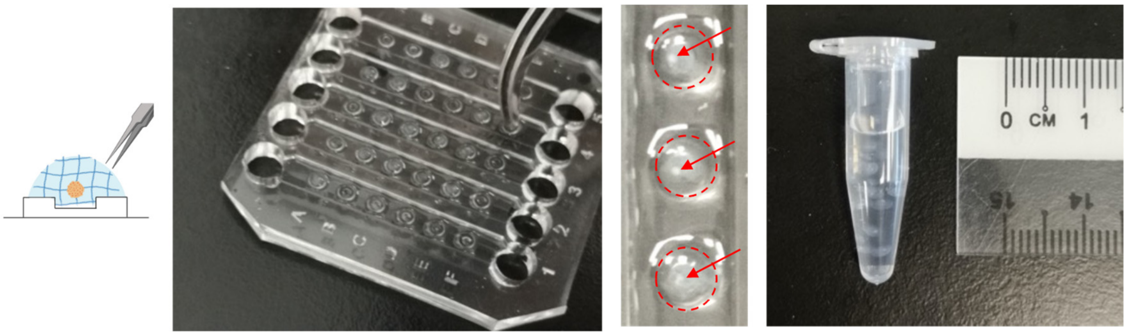

Figure S13. Retrieval of spheroid-laden hydrogel using forceps and resuspension in a microtube. Spheroid (small white dots) can be observed with naked eyes and are highlighted with red arrows.

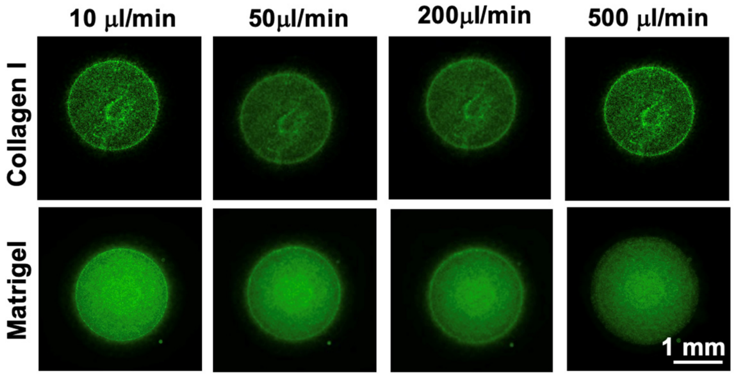

Figure S14. Stability of Collagen I (3 mg/mL) and Matrigel (4 mg/mL) at various flow rate. Hydrogel were mixed with fluorescent microbeads for visualization. Images were taken after 5 min of perfusion.
